# Chemotherapy (Etoposide)-Induced Intermingling of Heterochromatin and Euchromatin Compartments in Senescent PA-1 Embryonal Carcinoma Cells

**DOI:** 10.3390/cancers17152480

**Published:** 2025-07-26

**Authors:** Marc Bayer, Jaroslava Zajakina, Myriam Schäfer, Kristine Salmina, Felikss Rumnieks, Juris Jansons, Felix Bestvater, Reet Kurg, Jekaterina Erenpreisa, Michael Hausmann

**Affiliations:** 1 Kirchhoff-Institute for Physics, Heidelberg University, Im Neuenheimer Feld 227, 69120 Heidelberg, Germany; marc.bayer@stud.uni-heidelberg.de (M.B.); jaroslava@magisters.net (J.Z.); myriam.schaefer@kip.uni-heidelberg.de (M.S.); 2 Latvian Biomedical Research and Study Centre, Rātsupītes iela 1, LV-1067 Rīga, Latvia; kristine@biomed.lu.lv (K.S.); felikss.rumnieks@biomed.lu.lv (F.R.); jansons@biomed.lu.lv (J.J.); katrina@biomed.lu.lv (J.E.); 3 German Cancer Research Center (DKFZ), Im Neuenheimer Feld 280, 69120 Heidelberg, Germany; f.bestvater@dkfz-heidelberg.de; 4 Institute of Technology, University of Tartu, Nooruse 1, 50411 Tartu, Estonia; reet.kurg@ut.ee

**Keywords:** DNA damage by etoposide, chromatin re-organization in tumor cells, cell-fate, senescence, stemness self-renewal, single-molecule localization microscopy, statistical and topological data evaluation

## Abstract

**Background**: Often, neoadjuvant therapy, which relies on the induction of double-strand breaks (DSBs), is used prior to surgery to shrink tumors by inducing cancer cell apoptosis. However, recent studies have suggested that this treatment may also induce a fluctuating state between senescence and stemness in PA-1 embryonal carcinoma cells, potentially affecting therapeutic outcomes. Thus, the respective epigenetic pathways are up or downregulated over a time period of days. These fluctuations go hand in hand with changes in spatial DNA organization. **Methods**: By means of Single-Molecule Localization Microscopy in combination with mathematical evaluation tools for pointillist data sets, we investigated the organization of euchromatin and heterochromatin at the nanoscale on the third and fifth day after etoposide treatment. **Results**: Using fluorescently labeled antibodies against H3K9me3 (heterochromatin tri-methylation sites) and H3K4me3 (euchromatin tri-methylation sites), we found that the induction of DSBs led to the de-condensation of heterochromatin and compaction of euchromatin, with a peak effect on day 3 after the treatment. On day 3, we also observed the co-localization of euchromatin and heterochromatin, which have marks that usually occur in exclusive low-overlapping network-like compartments. The evaluation of the SMLM data using topological tools (persistent homology and persistent imaging) and principal component analysis, as well as the confocal microscopy analysis of H3K9me3- and H3K4me3-stained PA-1 cells, supported the findings that distinct shifts in euchromatin and heterochromatin organization took place in a subpopulation of these cells during the days after the treatment. Furthermore, by means of flow cytometry, it was shown that the rearrangements in chromatin organization coincided with the simultaneous upregulation of the stemness promotors OCT4A and SOX2 and senescence promotors p21Cip1 and p27. **Conclusions**: Our findings suggest potential applications to improve cancer therapy by inhibiting chromatin remodeling and preventing therapy-induced senescence.

## Simple Summary

Current cancer chemotherapy uses etoposide to induce DNA double-strand breaks for cell killing, which can cause fluctuations in stemness promoters like OCT4A, SOX-2, or NANOG in relation to senescence promoters p21Cip1, p27, or p16ink4A, and mitigate anti-cancer therapy. These fluctuations take place over several days and are associated with changes in the organization of euchromatin and heterochromatin. Here, we show how the induction of double-strand breaks by etoposide led to the compaction of euchromatin and de-condensation of heterochromatin on day 3. This was accompanied by a co-localization of euchromatin and heterochromatin marks as detected by Single-Molecule Localization Microscopy.

## 1. Introduction

Beyond the application of radiotherapy, a common way to treat cancer is to damage the DNA of cancer cells with chemotherapeutics such as etoposide (ETOs) [1,2,3]. ETO is used for the treatment of a number of cancer types including testicular cancer [4], lung cancer [5], lymphoma [6], leukemia [7,8], neuroblastoma [9,10], and ovarian cancer [11,12]. This drug inhibits the ligation activity of the topoisomerase II enzyme, which normally induces the relaxation of supercoiled DNA. As a result, it leads to double-strand breaks (DSBs) in the DNA of tumor cells, inhibiting DNA proliferation and forcing the cells to undergo apoptosis [13]. However, cancer cells often evade apoptosis and enter a different cell state called senescence [14,15]. In this state, the cells usually persist in cell cycle arrest as a cellular response to DNA damage [16]. Currently, many mechanisms behind senescence are insufficiently understood [17]. Until recently, it was unknown that senescence is involved in cases poorly responsive to neoadjuvant therapy [18]. Moreover, it has been found that senescence promotes in vivo reprogramming [9,10,11,12,13,14,15,16,17,18,19,20]. ETO treatment in PA-1 cells [21] can cause a paradox between self-renewal and senescence. Induced DNA damage causes the p53-dependent coupling of self-renewal and senescence pathways. Cells with sufficiently repaired DNA can enter mitosis while cells with persisting damage remain at late-S-G2M arrest and senescence [22,23]. p53 reduction, for instance, prevents the upregulation of OCT4A and p21Cip1, and senescence remains enhanced in combination with a loss of genome integrity. On the other hand, p53 enhancement safeguards DNA integrity and provides genome stability and self-renewal [22,23]. This is associated with OCT4A and p21Cip1 upregulation and changes in cell cycle regulation. Over several days, the cells gain an ability to adapt to a stemness and restart their proliferation [23]. In other words, cells must not persist in senescence and have the chance to escape into a self-renewal state [22,23], and this mechanism is conserved from cnidaria [20].

As the cell nucleus is a self-organizing system [24] that should react to any epigenetic changes via the response of chromatin reorganization, it has been assumed that epigenetic fluctuation, such as that described above, may be associated with changes in chromatin reorganization [25]. Although epigenetic fluctuations have been shown through molecular biological methods, such as flow cytometry and epifluorescence microscopy in detail in [18,22,23], so far, the microscopic quantification of euchromatin and heterochromatin development during the time course of fluctuation between senescence and stemness (days three to five after ETO treatment) is missing.

In this article, we used confocal microscopy and Single-Molecule Localization Microscopy (SMLM) [26,27,28,29,30,31,32], in combination with mathematical evaluation tools of statistics and topology [33], to study chromatin organization in the embryonal PA-1 teratocarcinoma cell line treated with etoposide [34]. SMLM in combination with Ripley statistics [35], persistent homology [36,37], persistent imaging [38], and after principal component analysis (PCA) [39] has been successfully applied to discriminate cell types according to their chromatin organization [33,40] and measure radiation treatment effects on heterochromatin [41].

The cells were treated with ETO-inducing DSBs [1]. Heterochromatin, a transcriptionally silent chromatin, was marked with anti-H3K9me3 antibodies [42] while transcriptionally active euchromatin was marked with anti-H3K4me3 antibodies [43]. In non-treated control specimens, both components are compartmentalized, while after ETO treatment, we identified the spatial dissemination of heterochromatin as well as an increase in euchromatin density; thereby heterochromatin–euchromatin co-localization was found to be a topological feature of senescence and self-renewal bi-potentiality.

To further control the stemness and senescence induction after DNA damage, flow cytometry was conducted, as described in detail in [23], and it confirmed that ETO treatment also induced the overexpression of OCT4A and SOX2 stemness markers as well as p21Cip1 and p27 senescence markers in PA-1 cells.

## 2. Materials and Methods

### 2.1. Cell Line and Cell Preparation

A PA-1 cell line [21,34] was derived from the ascitic fluid cells of a 12-year-old Caucasian female with a teratocarcinoma of the ovary. The cells were characterized by wild-type p53, diploidy, and a stable karyotype with one permanent translocation. The PA-1 cells (ATCC, Manassas, VA, USA) were cultivated in Dulbecco’s Modified Eagle Medium (DMEM) containing 10% fetal bovine serum (FBS, Sigma, St. Louis, MO, USA). The cells were maintained in a 5% CO incubator at 37 °C without antibiotics. During the exponential growth phase, they were treated with 8 µM etoposide (ETO) for 20 h. Following drug removal, the culture medium was refreshed every 48 h, and cells were collected for analysis on days 3 and 5 after ETO treatment. As shown previously [22], a proportion of cells survived this treatment and restored the clonogenic growth from day 7. In parallel, non-treated (NT) control cells were cultured. Cytospins were prepared as described in detail elsewhere [44]. The cells were fixed in cold methanol followed by three quick dips in acetone before they were labeled using the following antibody system: As the primary antibody, the mouse monoclonal anti-H3K4me3 (ab1012, Abcam, Cambridge, UK) [45] was used for specific euchromatin staining. According to the manufacturer, this antibody does not cross-react with unmodified histone H3 or with mono-, di-, tri-methyl K9, di- or tri-methyl K27 peptides. As the second primary antibody, the rabbit polyclonal anti-H3K9me3 (ab8898, Abcam) [46] was used for specific heterochromatin staining. It shows low cross-reactivity with tri-methyl K27, which shares a similar epitope, and does not bind to mono- or di-methylated K9. Additionally, the antibody was ENCODE-validated [47,48] and showed high specificity and no cross-reaction in a dot blot analysis. The following combination of secondary antibodies was used: Euchromatin was stained with a goat anti-mouse IgG (H + L) AlexaFluor488 cross-adsorbed antibody (A11001, Invitrogen, Thermo Fisher Scientific, Waltham, MA, USA) and heterochromatin with a goat anti-rabbit IgG (H + L) AlexaFluor594 highly cross-adsorbed against mouse IgG antibody (A11037, Invitrogen). Alexa Fluor 488 (excitation/emission maximum 499/520 nm) and Alexa Fluor 594 (excitation/emission maximum 590/618 nm) have well-separated spectra; therefore, the overlap between their emission windows was negligible using appropriate filter settings in the instrumentation. The cell nuclei were counterstained with DAPI.

### 2.2. Confocal Microscopy

Samples were analyzed with a confocal laser scanning microscope LSM710 (Carl Zeiss AG, Oberkochen, Baden-Württemberg, Germany), using a 63×/NA1.3 objective (Carl Zeiss AG) with appropriate filter settings for the Alexa dyes used (see Section 2.1). The images obtained were analyzed with the commercially available ZEN2011 software (blue edition) (Carl Zeiss AG).

### 2.3. Single-Molecule Localization Microscopy (SMLM)

SMLM makes use of the stochastic blinking of dye molecules for the precise localization of single antibodies. Dye molecules stochastically switch between two states: fluorescing and non-fluorescing. The SMLM setup is described in detail in [33]. For the acquisition of the data sets used in this article, the 405 nm laser (120 mW) was used for DAPI widefield control. For the euchromatin and heterochromatin marks, the 491 nm (200 mW) and 568 nm (200 mW) illumination lasers were used. Blinking events were recorded using an Andor Ultra EMCCD (Oxford Instruments, Tubney Woods, Abingdon, Oxfordshire, UK) camera via appropriate filter settings.

The settings for heterochromatin imaging were an EMCCD gain of 120, 30 ms exposure time, 561 nm laser at 80% power, and flash at 90% for 20,000 ms, i.e., for 4000 frames. Only the last 2000 frames were used for analysis, since the first 2000 frames showed signal overexposure. The settings for euchromatin imaging were an EMCCD gain of 120, 30 ms exposure time, 491 nm laser at 80% power, and flash at 80% for 10,000 ms, i.e., for 2030 frames. Here, the first 30 frames were not considered for the analysis because of possible signal overexposure. These settings made photo-bleaching (a shortcoming in SMLM) negligible. Crosstalk between the two fluorescence channels was considerably reduced by thresholding during data acquisition.

The software Omicron and Live Acquisition (Carl Zeiss AG) were used for standard microscopy imaging.

Non-apoptotic cells, fitting within the microscope’s field of view, and single nuclei without visible nonspecific fluorescent aggregates were selected for SMLM data acquisition. This excluded a considerable amount of cell nuclei, especially in the cases after ETO treatment. However, such exclusion appeared to be acceptable since not all cells reacted synchronically. Therefore, only those cells that were homogeneous in size and shape were picked out.

### 2.4. Data Processing of SMLM Data Sets

Using an in-house software package [33], super-resolution signal coordinates were calculated according to an algorithm subtracting the brightness values of two successive image frames. Dark states over more than two successive frames were registered, and the barycenters were calculated [49]. Finally, the coordinates and localization precision of the loci of all blinking molecules were registered in the “orte matrix”, which was used as the basis for further mathematical calculations [33]. In total, 16 nuclei were evaluated for the non-treated sample on day three, and 25 nuclei were evaluated for the non-treated sample on day five. In the case of the ETO-treated specimens, 45 nuclei were evaluated each for day three and day five.

For the one-channel analysis, Ripley statistics [35] were applied to the coordinates of the points, and point-to-point distances were calculated and normalized. The peaks in Ripley distance frequency histograms indicate point clustering. For topological analysis, persistent homology [36,37], persistent imaging [38], and principal component analysis [39] were applied as described in detail in [33]. A transformation into the topological space was obtained by two parameters: the number of “components” and number of “holes” in the detected point pattern. Each component was represented by a bar starting at 0 (i.e., the given point) and ending at the radius value, where two artificial increasing circles around the points were attached and the two components merged into one. Since enlarged components could arise, forming a closed structure with “free” space inside uncovered by the circles, holes were born. If such a hole was completely covered by the increasing virtual circles, this bar also ended up as a hole. Using this process, the point pattern recorded was transferred in an equivalent bar code pattern [33,36,37].

The bar codes of the holes were transferred into a graph of “bar lifetime” vs. “bar birth”. Then, this graph was overlaid by a pixel scan of equally sized pixels. This is called a persistent image [38]. In this persistent image, the intensity of each pixel directly correlates to the number of points in the given pixel. In this way, persistent images were created for each cell nucleus and each type of labeling. These persistent image values were finally transferred into an n-dimensional orthogonal vector space. Pixel by pixel, the intensity values were transferred into this vector space, i.e., all values of the 1st pixels were transferred in the first dimension; all values of the 2nd pixels were transferred in the second dimension; and so on. Finally, the result was subjected to principal component analysis (PCA) [39], by which the multi-dimensional feature space was reduced to the 2D latent space of the two largest variations in topology. Thus, only two principal components describe the main features and their changes. Small variations (“biological noise”), i.e., the other higher dimensions, were neglected.

For cases involving the co-localization of two signals, it was considered that SMLM works on the level of single molecules that do not intermingle. This means that co-localization is not visible by an overlay of two colors as a fused mixed color (for instance, where red and green co-localizing signals form a yellow one). Based on the size of the antibodies, co-localization in SMLM is defined by a minimum distance calculated on the basis of antibody size and localization precision [50]. A typical IgG antibody has a size of about 14.5 × 8.5 × 4 nm^3^. The secondary antibody can be oriented at different angles relative to the main axis of the primary antibody. If we assume an angle of 90°, two fluorescence molecules bound to two neighboring antibody constructs would have the following maximum geometrical distance:

2 × (4 nm + 8.5 nm + 14.5 nm) = 54 nm

It is also assumed that each fluorescence molecule is typically detected with a localization precision of about 20 nm and 40 nm in a two-color experiment. Thus, the limit of co-localization is defined as follows: “Two fluorescence molecules are assumed to co-localize, if their distance is <95 nm”.

### 2.5. Flow Cytometry

For flow cytometry, PA-1 cells were harvested at the indicated time points and washed with PBS. Cells were then fixed with BD Cytofix Fixation Buffer and permeabilized with BD Phosflow Perm Buffer III (Becton Dickinson – BD Biosciences, Customer Service Interlux, SIA, Stopiņu novads, Latvia), as recommended by the manufacturer. Cells were incubated with the anti-Oct3/4 AlexaFluor647-conjugated antibody (Santa Cruz Biotechnology; Dallas, TX, USA), Catalog No sc-5279 AF647), anti-Sox2 V450 (BD Horizon™, Becton Dickinson – BD Biosciences, Catalog No 561610), anti-p21/CIP/CDKN1A AlexaFluor 750-conjugated antibody (R&D Systems; Minneapolis, MN, USA), Catalog No IC1047S-100UG), the p27/Kip1 Alexa Fluor 488-conjugated antibody (R&D Systems, Catalog No IC2256G-100UG), and the PE mouse anti-γH2AX (BD Biosciences; Franklin Lakes, NJ, USA, Catalog No 562377), all diluted in BD Pharmingen Stain Buffer for 30 min at +4 °C. Flow cytometry was performed on a FACSAria II cytometer (Becton Dickinson - BD Biosciences), using the 633 nm laser to detect SOX2 and p27, with APC and APC-Cy7 fluorescence channels, respectively. The 488 nm laser was used to detect γH2AX in the PE fluorescence channel. We used FACSDiva v. 6.1.3 software (BD Biosciences) on the flow cytometer and analyzed the data using the FlowJo v10 software (BD Biosciences).

## 3. Results

To investigate the spatial organization of euchromatin and heterochromatin, the PA-1 teratocarcinoma cells were treated with etoposide, cultured for three and five days, cytospun onto glass slides, fixed in cold methanol with short dips in acetone, and stained with anti-H3K9me3 (heterochromatin) and anti-H3K4me3 (euchromatin) antibodies as described above. The controls were not treated with etoposide but cultured for three and five days in parallel.

**Figure 1:**
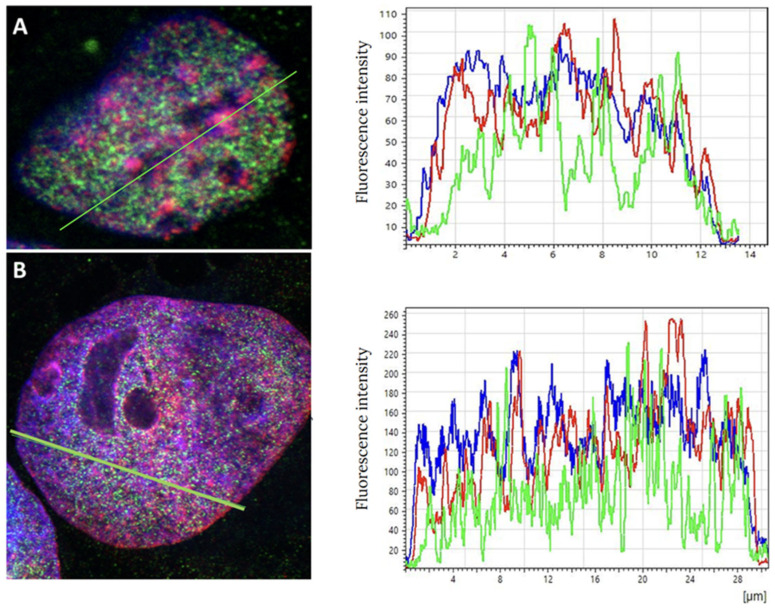
Examples of confocal images of PA-1 cell nuclei. (**A**) A non-treated cell nucleus. The intensity lines along the green line in the image are shown on the right for this given cell nucleus: heterochromatin (red curve) shows increased density values where euchromatin (green curve) is reduced and vice versa. The blue curve shows the DAPI intensity, which represents chromatin density and is independent of the differentiation of hetero- and euchromatin. (**B**) An ETO-treated cell nucleus (day three). The intensity lines along the green line in the image are shown on the right for this given cell nucleus: heterochromatin (red curve) and euchromatin (green curve) maxima are not always separated or opposed, indicating an intermingling of heterochromatin and euchromatin in at least one part of the cell nucleus. The blue curve shows the DAPI intensity, which represents chromatin density and is independent of the differentiation of hetero- and euchromatin.

To examine the compartmentalization of heterochromatin and euchromatin, confocal microscopy was applied. In Figure 1, two typical examples for a treated and a non-treated nucleus are shown. Heterochromatin and euchromatin seem to be strictly separated from each other in the non-treated control sample (Figure 1A). The intensity curves indicate a clustering of heterochromatin in the regions of low euchromatin and vice versa, while the DAPI intensity curve represents a fluctuating DNA distribution. It is clear that heterochromatin is localized around NORs. Further images are shown in the Supplementary Material (Figure S1).

After the ETO treatment on day three, heterochromatin seems to be fragmented (smaller peak structures) and intermingles with euchromatin (Figure 1B). This loss of compartmentation between heterochromatin and euchromatin supports the assumption that the ETO treatment may cause the pulsing of heterochromatin into euchromatin regions. In addition, euchromatin in part seems to surround NORs.

In order to further study the effects of ETO treatment in more detail and to quantitatively measure this for the first time, further specimens were analyzed using super-resolution SMLM. After optimizing the detection parameters for the given specimens, super-resolution SMLM was performed for non-treated and ETO-treated cell nuclei on days three and five. The data were quantitatively evaluated [33] by means of Ripley statistics and DBScan clustering evaluation. The results of each color were evaluated separately using the in-house-made program tools described above. For the identification of the molecule clusters in euchromatin and heterochromatin using DBScan, the minimum numbers of points located within a minimum cluster radius of 200 nm were chosen. These values were chosen by considering the concentration of fluorophores on the slides and blinking events measured (Table 1).

**Figure 2:**
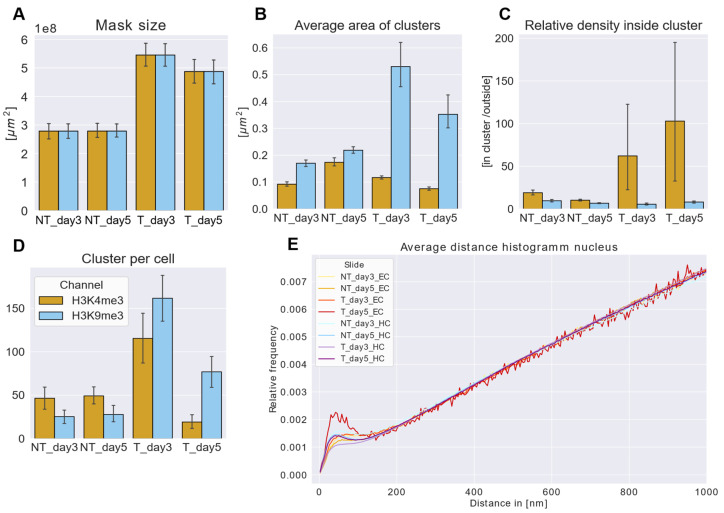
Statistics of euchromatin and heterochromatin organization in PA-1 cells and their changes after etoposide treatment. Euchromatin (EC), marked by H3K4me3 (yellow), and heterochromatin (HC), marked by H3K9me3 (blue), were analyzed under different conditions: non-treated (NT) on days 3 and 5, and etoposide-treated (T or ETO) on days 3 and 5. (**A**) The mask size represents the total nuclear area of PA-1 cells. (**B**) The average cluster area for euchromatin and heterochromatin. (**C**) The relative density inside chromatin clusters. (**D**) The number of chromatin clusters per cell. (**E**) The distance frequency histogram of the pairwise distances between measured signals within nuclei (Ripley statistics). The peaks on the left represent cluster formation at a size of about 160 nm in diameter. The follow-up linear increase is compatible with random distribution. Error bars in (**A**–**D**) represent the SD.

In Figure 2, the statistical data are summarized: A clear difference in the size of the nuclei (provided by the mask size) of the ETO-treated and non-treated cell nuclei was found (Figure 2A). ETO-treated cells showcased an increase in their nuclear size on both analyzed days compared to non-treated control samples cultivated until the same day. This increase was more pronounced on day three than on day five. This effect was true for cells of both types of chromatin labeling. This result might be biased in some way because only cell nuclei that showed an average size with some variability were chosen for SMLM, while extremely big (often polyploid) examples were excluded from the evaluation due to limitations of the microscope’s field of view.

Clusters of labeling points were determined according to the parameters described in Table 1. The ETO-treated cells displayed a steep increase in the average cluster area of the heterochromatin clusters and reached a value that was multiple times higher than the area of euchromatin clusters (Figure 2B). In contrast to heterochromatin, the average area of euchromatin clusters on day three was comparable to that of the non-treated samples. Moreover, on day five, the average area with euchromatin clusters was even lower. For both heterochromatin and euchromatin, a cluster size decrease was observed between day three and day five. During this time period, the area of euchromatin clusters dropped below the level of non-treated cells on day three.

The relative signal density inside the clusters (the amount of signals detected inside the given clusters compared to those outside of them) showed constant low values for heterochromatin in treated and non-treated samples (Figure 2C). In contrast, there were changes in the relative signal density of euchromatin inside the clusters in the ETO-treated samples. A considerable rise was noticed on day three, which continued to increase on day five. However, these values showed a large SD, i.e., the numbers between different cell nuclei showed a large variation. This indicates different activities of individual cell nuclei. Nevertheless, the data across all cells that were measured indicate that euchromatin formed variable clusters during treatment.

Along with the increase in the cluster areas (Figure 2B), the number of clusters per cell also showed a significant increase for the treated cells. This increase reached its maximum peak on day three and decreased again on day five (Figure 2D). Here, an interesting trend was revealed: the non-treated samples always showed a higher number of euchromatin clusters than heterochromatin clusters. At first glance, this might be reasoned by the labeling efficiency of the antibodies used. However, this did not seem to explain the effect, because this trend was reversed for the treated cells. Here, a higher number of heterochromatin clusters than euchromatin clusters was found for both day three and five. Also, a significant decrease was observed between day three and day five for both euchromatin and heterochromatin; thus, the amount of euchromatin dropped significantly below the level of the NT cells (Figure 2D).

Furthermore, the acquired data were used to create a relative pairwise distance frequency histogram (Ripley curve). From such Ripley curves, information about the structures formed by the chromatin was extracted (Figure 2E). The Ripley curves showed distinct peaks in the regime of 20 nm to 160 nm for all samples, reflecting the presence of some non-random structures in a random environment. All evaluated slides showed a similar behavior in the formation of structures with two slightly deviating exceptions: heterochromatin on day three after ETO treatment had the most relaxed organization, which was more compacted on day five, while euchromatin on day five showed the most clusters. Heterochromatin on day three showed a lower and more spread-out accumulation of clusters, lacking a distinctive peak. This indicated a change in chromatin structures using chromatin fluctuations. This demonstrates a more prominent formation of structure and, thus, organization of the euchromatin unusual under normal (proliferating) cell conditions.

**Figure 3:**
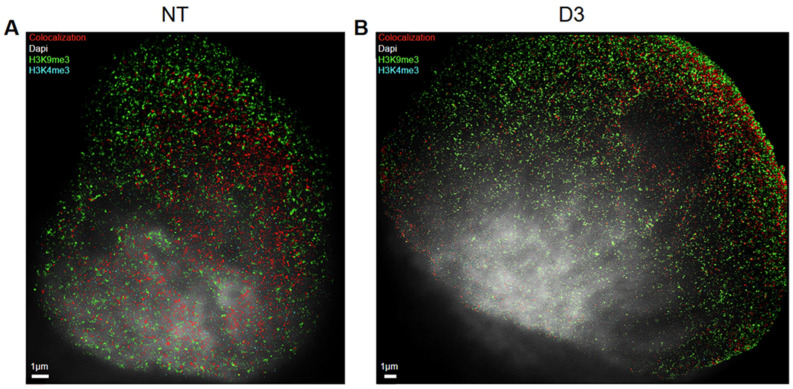
Co-localization example images of a non-treated (NT) and ETO-treated cell nucleus: red signals represent co-localization between euchromatin and heterochromatin tags (distance ≤ 95 nm; for further explanation, see the Materials and Methods); blue signals represent euchromatin (anti-H3K4me3 labeling); and green signals represent heterochromatin (anti-H3K9me3 labeling). (**A**) Co-localization in a non-treated cell nucleus (NT) on day five and (**B**) co-localization in a treated cell on day three (D3).

The two-channel analysis program was used to further extrapolate information about the co-localization of heterochromatin and euchromatin. In contrast to standard light microscopy, where two “co-localizing” red and green signals form a yellow one, co-localization in SMLM means that the distances between the two signals are ≤ 95 nm. The 95 nm value was chosen as a threshold based on the extrapolated data and considering the distance structure of the heterochromatin and euchromatin antibody constructs. This means, for physical reasons, that two antibodies can never occupy the same place simultaneously. Thus, this value considers the size of the two labeling complexes of primary and secondary antibodies (see [50] for details). From the extracted SMLM data (“orte” matrix), co-localization overview images were created in order to obtain a visual impression of how organized and non-organized chromatin looks (Figure 3). Here, it is also noteworthy that in the non-treated control cells, euchromatin tended to be located in the middle of the nucleus surrounded by heterochromatin (Figure 3A), as expected under usual conditions. After the ETO treatment, however, euchromatin was often found near the nuclear membrane (Figure 3B) while heterochromatin was found everywhere, including the nuclear center. It seemed to be co-localizing everywhere in the nucleus, especially at the nuclear boundary, instead of being focused on the center.

**Figure 4:**
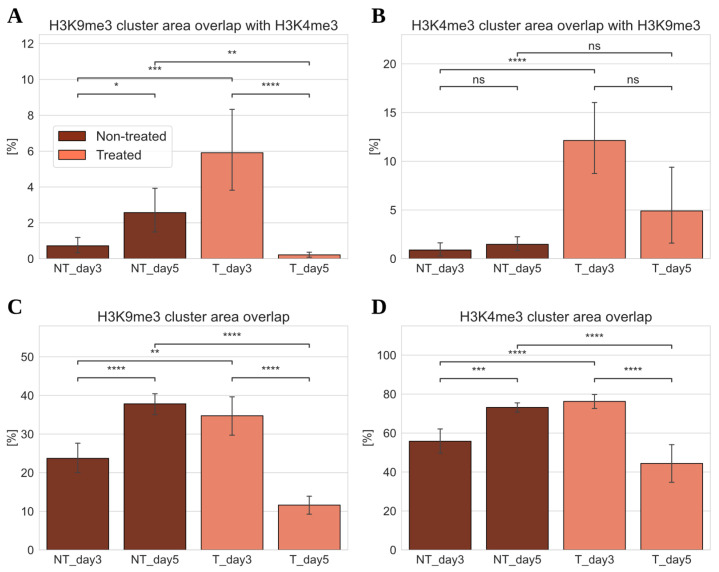
The percentage of overlap in the cluster area between heterochromatin (HC) and euchromatin (EC) in ETO-treated (T) cells in comparison to non-treated (NT) cells. Error bars show the standard deviation. The degree of statistical significance is given by *, **, ***, ****. n.s.: not statistically significant. (**A**) The percentage of H3K9me3 clusters (HC) that overlap with H3K4me3 clusters (EC). (**B**) The percentage of H3K4me3 clusters (EC) that overlap with H3K9me3 clusters (HC). (**C**) The percentage of H3K9me3 fluorescent signals (HC) in H3K4me3 clusters (EC) that co-localize with H3K4me3 (EC) fluorescent signals. (**D**) The percentage of H3K4me3 fluorescent signals (EC) in H3K9me3 clusters (HC) that co-localize with H3K9me3 fluorescent signals (HC). Note that all figures have different scaling.

Euchromatin (anti-H3K4me3 tags) co-localized most strongly with heterochromatin (anti-H3K9me3 tags) and vice versa on day three after ETO treatment (Figure 4). Here, the euchromatin clusters cover 5% (with high variability) of the heterochromatin cluster areas (Figure 4A), while the heterochromatin clusters cover approximately 30% of the euchromatin cluster areas (Figure 4B). In contrast, on day five post-ETO treatment, there was nearly no co-localization between the two cluster types. Furthermore, the data also showed little co-localization for the NT cells, especially on day five. Only on day three after ETO treatment did a significant co-localization between hetero- and euchromatin occur.

Also, the percentage of the two tags, anti-H3K4me3 and anti-H3K4me3, that co-localized with the signals within the clusters of their respective counterparts was calculated. Regarding co-localization within euchromatin clusters (Figure 4C) and within heterochromatin clusters (Figure 4D), it was found that the non-treated controls on day five and the ETO-treated cells on day three showed a significantly higher co-localization of signals compared to those on day five post-ETO treatment. However, only about half as many H3K4me3 signals co-localized within euchromatin clusters compared to H3K4me3 in heterochromatin clusters. Furthermore, co-localization on day three after ETO treatment was slightly lower (or equal within the error margins) to that of the day-five control, with day five post-treatment showing the lowest value.

For both, the non-treated controls on day five and the ETO-treated cells on day three, most euchromatin signals co-localized with the cluster signals of heterochromatin (Figure 4D). On day five after ETO treatment, the co-localization of these signals dropped significantly and reached the lowest values. This showed that the distances between the different tags increased compared to the other samples. This indicated a reduced interaction between the structures marked by these two tags. On day three of post-ETO treatment, nearly every euchromatin signal within the heterochromatin clusters was close enough to co-localize with them. In constrast, on day five, the opposite seemed to be true (compare Figure 4C and Figure 4D).

In summary, the data indicate **a significant overlap between hetero- and euchromatin clusters on day three post-ETO treatment**, while this was nearly absent on day five. Furthermore, in general, the co-localization of H3K4me3- (euchromatin) tags in heterochromatin clusters was about twice as likely as the reverse. This suggests that euchromatin and heterochromatin were closer together within the heterochromatin clusters than within euchromatin clusters. By day five post-ETO treatment, co-localization with the signals of the other channel was significantly reduced. This indicated that even if these signals were within a cluster of the other type, they were farther apart from each other compared to day three.

**Figure 5:**
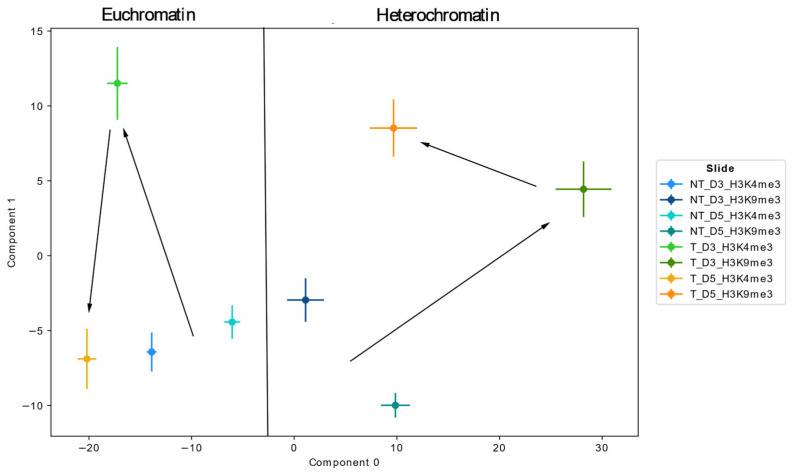
Principal component analysis (PCA) of the chromatin topology of ETO-treated PA-1 cells in comparison to non-treated control cells. Component 0 in the latent space represents a clear distinction between euchromatin and heterochromatin features, while component 1 indicates the fluctuation of euchromatin (day 3 to day 5 vs. control) and heterochromatin (day 3 and day 5 vs. control) of the ETO-treated samples during the time period of days three and five. The differences in the non-treated control cells might be due to chromatin organization changes during the cell cycle.

The topological properties of the examined chromatin organization were identified after persistent homology and imaging using PCA on the images of the holes (Figure 5). The latent space of the first two components (0 and 1) revealed basic changes in the organizational structures of H3K4me3 tags and H3K9me3 tags, i.e., of eu- and heterochromatin, which differed fundamentally along the 0-component. This already demonstrated a fundamental difference in the topological properties between eu- and heterochromatin across all examined samples, including the controls.

The PCA of the two controls showed that they share similar properties in the case of H3K4me3, as indicated by their close proximity in component 1 in the latent space. In contrast to the non-treated controls, the values of the ETO-treated samples deviated significantly from the non-treated controls on day 3. For euchromatin, the data showed a significant change in component 1 on day three, returning to a state comparable to the NT controls on day five. Euchromatin only deviated slightly in component 0 for both measured time points post-ETO treatment.

For heterochromatin, on day three, the ETO treatment led to a substantial change in both component 0 and component 1, representing a greater deviation from the topological properties of the non-treated controls, as can be observed for euchromatin. On day five, the deviation in component 0 was reversed, but a topological state close to the non-treated controls was not fully approached. Component 1 deviated even more from the non-treated controls than on day three post-ETO treatment. Considering the development of these values, it was concluded that euchromatin changed its structural properties on day three and returned to a state close to its original non-treated state, even though it deviated slightly. Compared to this, heterochromatin underwent a drastic change regarding its structure on day three and did not return to its original properties on day five.

**Figure 6:**
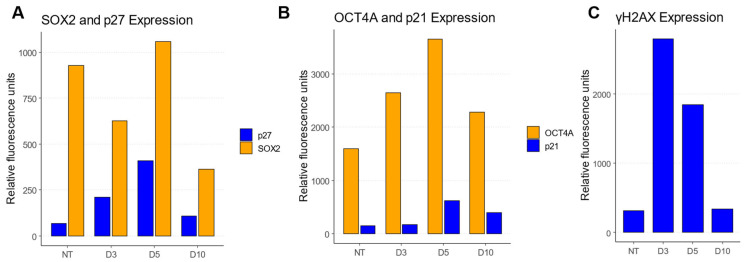
The flow cytometry of cell cycle checkpoint regulator expression after ETO treatment. (**A**) Analysis of SOX2 and p27 on days three, five, and ten after ETO treatment compared to the non-treated (NT) control. (**B**) Analysis of OCT4A and p21Cip1 on days three, five, and ten after ETO treatment compared to the non-treated (NT) control. (Note: The abscissa in (**A**,**B**) is given in relative fluorescence since a flow cytometer does not measure absolute intensity values). (**C**) Analysis of γH2AX expression in ETO-treated cells on days three, five, and ten compared to the non-treated (NT) control, indicating a considerable amount of double-strand breaks after ETO treatment. The number of single PA-1 cells used for analysis was between 50,409 and 296,329 per experiment. Since detailed flow cytometry measurements have been published elsewhere [22,23], the experiments were only conducted once.

To check the balance of the expression of the drivers behind senescence (p27 and p21Cip1) and stemness (SOX2 and OCT4A) in ETO-treated cells, flow cytometry was performed (Figure 6A,B). Both senescence drivers p27 and p21Cip1 were upregulated after ETO treatment. The stemness driver OCT4A was also upregulated following ETO treatment. Conversely, another stemness driver, SOX2 expression, despite being upregulated, shrank due to inhibition from p27, while being overexpressed on day five. On day ten, all the factors were reversed close to their original control state with the exception of SOX2, which was downregulated in comparison to its control state.

The γH2AX expression increased on day three after ETO treatment and decreased by one third on day five and finally back to the control level on day 10 (Figure 6C). This may have indicated the occurrence of double-strand breaks (DSBs) on day three and a running repair over day five up to day ten. Compared to the trend observed for hetero- and euchromatin, these results indicate similar behavior. When organizational changes started to reverse to a control-like state between day three and five, corresponding to a return to the G1 state in a proportion of cells, the release of G2M arrest and restarting of the cell cycle might occur [23]. Another possibility behind the reduction in γH2AX expression might be that instead of γH2AX, H2AJ formation took place, which has been described recently as an effect of senescence [17].

## 4. Discussion

Recently, it has been described that cellular senescence occurring in development and under different kinds of stress, including cancer, chemo-radiotherapy, can promote in vitro and in vivo reprogramming [9,10,11,12,13,14,15,16,17,18,19,20]. The induction of DSBs either chemically or by irradiation could be the reason for the paradox between self-renewal and senescence, where cells are able to again enter mitosis or remain at the G2/M checkpoint, which prompts either state [23]. It was also shown that a p53 dependency on OCT4A (the stemness driver) and p21Cip1 (senescence driver) exists [14,15,19]. Furthermore, it was revealed that this is associated with p53 and its induced OCT4A and p21Cip1 competitive upregulation enables the cells to adapt to a stemness state and restart their proliferation [22].

Using SMLM and novel data computing tools [26,33], distinct and reversible changes in the organization of euchromatin and heterochromatin were observed in ETO-treated PA-1 cells, reflecting the role of H3K9me3 heterochromatin in the establishment and maintenance of cell identity and the regulation of gene expression through the silencing position variegation effect [25,43]. This was seen in response to ETO-mediated DNA damage, where cellular senescence was also previously seen on days three to five via the sa-β-galactosidase reaction [22]. ETO-induced DSBs, as indicated by an increase in γH2AX, led to significant heterochromatin relaxation and dissemination. In contrast, euchromatin showed an increase in density and condensation. On day three after treatment, heterochromatin showed a dispersed structure, indicated by increased cluster areas and reduced cluster density. This relaxation may influence the accessibility of repair proteins to DNA [25]. Such relaxation could be induced by the mechanical unfolding of heterochromatin, resulting from the release of folding tension after the introduction of DSBs.

Euchromatin condensation might be associated with transcriptional silencing, a protective response to prevent the transcription of damaged genes. Specifically, the de-methylation of heterochromatin, in particular that, which is nucleolar-associated, and the methylation of euchromatin, were found previously [44]. Euchromatin condensation peaked on day five and might suggest that transcription was suppressed. It may possibly be linked to the G2M cell-cycle arrest that might have taken place. This may cause the changes in the mask/nucleus size (see Figure 2A) and explain the observations of larger cells that were also described in previous studies [23]. It was found that the co-localization of H3K4me3 and H3K9me3 signals significantly increased on day three after ETO treatment, followed by a state below the level of the non-treated control state on day five.

This implies the much lower phase separation of euchromatin and heterochromatin on day three, in contrast to the strict phase separation that occurs on day five again. A possible cause for this co-localization is the dissemination of heterochromatin with a following overlap with euchromatin regions. For the presence of bivalent chromatin regions, further research may be required.

The process of epigenetic fluctuations is accompanied by a change in the eu- and heterochromatin distribution within the nuclei. This treatment induces the reversal of chromatin distribution. Euchromatin predominantly occupied the edge of the nuclei, leaving heterochromatin in the center. This stands in contrast to the distribution measured for normal cells and especially non-treated controls, where euchromatin can preferentially be found in the center of the nucleus. This relocation of euchromatin from the nucleus center to the edge reinforces the possibility that the transcriptional silencing of euchromatin might occur while accessibility to heterochromatin is enhanced. This might also indicate a conversion of euchromatin into facultative, inactive heterochromatin, where it might be compacted by the H3K27me3 histone state.

Flow cytometry showed the increased expression of both stemness and senescence markers after ETO treatment, which could be due to the transitional stage of the cells in which they oscillated between stemness and senescence fate decision [22,23]. γH2AX foci expectedly increased after ETO treatment, peaking on day 3 and declining by day five. This is typical for the successful repair of DNA damage in a proportion of cells, as shown earlier by the reduction in CHK2 signaling [22].

PCA highlighted topological differences, in general, between euchromatin and heterochromatin, as well as their responses to DNA damage. In contrast to heterochromatin, euchromatin returned in component 1 to a level close to the control on day five. Heterochromatin, however, still deviated, primarily in component 1, from the control on day five. Both euchromatin and heterochromatin deviated from the topological features of the controls on day three, with euchromatin mostly in component 1 and heterochromatin in components 0 and 1.

Regarding limitations and improvements, the SMLM analysis was limited to nuclei that fit within the microscope’s field of view, potentially introducing selection bias. The measurements produced by Single-Molecule Localization Microscopy (SMLM) are single-cell measurements. This means that only one cell is in the image frame that is acquired. Usually, whether on one or several slides, these single cells are visually preselected before data acquisition, since the data acquisition and evaluation per cell is time-consuming, so that only cells showing an appropriate preparation quality are finally subjected to the SMLM data acquisition. This means, for instance, that no bright background is allowed, the number of blinking events should be high enough for further computing, and the numbers of blinking events are in a comparable range, etc. Thus, from the beginning, the cells included had to fulfill some acquisition standards. Nevertheless, there is also considerable variability on one slide, which can be seen from the error bar by the number of clusters, which reflects the number of blinking events. A vast experience in SMLM single-cell experiments has shown that the difference between cells on one slide can be as large as the differences between the two slides. We are aware that this preselection may bias the results. But comparable procedures are, in general, applied in microscopy imaging to reduce the poor results obtained by limited preparation quality. Moreover, real biological reasons had to be taken into consideration. Recently, Alekseenko et al. [51] pointed out the reasons for the fundamentally low reproducibility of the quantitative results between individual cancer cells. For each cell that can be subjected to single-cell experiments, slightly different conditions in the microenvironment might lead to small functional differences in the individual cells; however, they all produce the same tumor or cell type. This might be negligible in bulk experiments; however, in single-cell measurements, it can be more prominent. In other words, the more precise single-cell measurements performed, the more individual variations may become obvious without using different experimental replicates.

Nonetheless, the findings show a mode of behavior in embryonic stem cells reflecting the reprogramming associated with considerable cellular senescence. Regarding this, our findings reveal the dual state and pulsing of heterochromatin both into and out of euchromatin, which, in turn, might imply the loss of heterochromatin silencing effect variegations throughout these fluctuations. The position-effect variegations observed show how heterochromatin packaging spreads across the transitional border between hetero- and euchromatin, which, in turn, causes transcriptional silencing in a stochastic pattern [52]. Although this study focuses on one cancer cell line only, these findings can be generalized for the nuclear regulation of other functions based on the structure in development. The use of multiple cell lines and advanced imaging techniques in future studies could make the conclusions more reliable and enable better understanding of chromatin dynamics. To study the bivalency of the chromatin after ETO treatment, other markers specific to bivalent chromatin, such as H3K27me3, could also be used in further research.

The presentation of chromatin re-organizations by SMLM and novel mathematical tools is, to our best knowledge, the first approach attempted to quantify chromatin changes over days on the nanoscale. The feasibility of such experiments offers new perspectives in senescence research as well as providing insight into position-effect variegation connected to the dual state of stemness and senescence for future investigations and further individualized cancer research.

## 5. Conclusions

Using SMLM in combination with novel mathematical tools for the first time to investigate stemness and senescence-related chromatin re-organization, this study revealed distinct structural responses of hetero- and euchromatin to ETO treatment. Heterochromatin was more disseminated after ETO treatment, whereas euchromatin became more condensed. Regarding the fact that we showed a fundamentally different chromatin distribution on day three post-ETO treatment, a lowered phase separation of hetero- and euchromatin was shown on day three, as indicated by an elevated co-localization rate and an increased phase separation of hetero- and euchromatin, followed on day five. These findings were accompanied by an increase in the opposite regulators OCT4, SOX2, and p27 with p21Cip1, which are the promoters for stemness and senescence. Furthermore, changes in the topological characteristics of both hetero- and euchromatin on day three were measured, and the different ways these characteristics were restored on day five for euchromatin and heterochromatin. In conclusion, the results indicate that the fluctuations in chromatin organization create the potential for considerable epigenetic changes, transferring the senescent state to the stemness state and vice versa.

## Figures and Tables

**Table 1:** Minimum number of points required to define a cluster for each object glass.

	NT Day 3	NT Day 5	ETO Day 3	ETO Day 5
**H3K4me3**	10	20	10	20
**H3K9me3**	40	80	60	80

## Data Availability

The data and software are part of the KIP SMLM data archive and can be obtained upon request from the corresponding author.

## Supplementary Materials

The following supporting information can be downloaded at https://www.mdpi.com/article/10.3390/cancers17152480/s1. Figure S1: Examples of ETO-treated and non-treated cell nuclei of PA-1 cells on day 3 and 5 after labelling of euchromatin with anti-H3K4me3 antibodies and heterochromatin with anti-H3K9me3 antibodies. While in the non-treated nuclei the chromatin typically shows a granular, network-like distribution, this is more dissolved in the treated cells indicating a loss of the strong separation of eu- and heterochromatin.

## Abbreviations

The following abbreviations are used in this manuscript:

HC	Heterochromatin
EC	Euchromatin
ETO	Etoposide
NT	Non-treated (cells)
T	Treated (cells)
SMLM	Single-Molecule Localization Microscopy
