# Supplementary material for: Chemotherapy (Etoposide)-Induced Intermingling of Heterochromatin and Euchromatin Compartments in Senescent PA-1 Embryonal Carcinoma Cells
Source: Cancers (Basel). 2025 Jul 26;17(15):2480. doi: 10.3390/cancers17152480 (PMC12345858; doi:10.3390/cancers17152480)
Supplement: Supplementary file 1 [file cancers-17-02480-s001.zip › cancers-3718259-supplementary.pdf]

cancers

MDPI

Supplementary Material

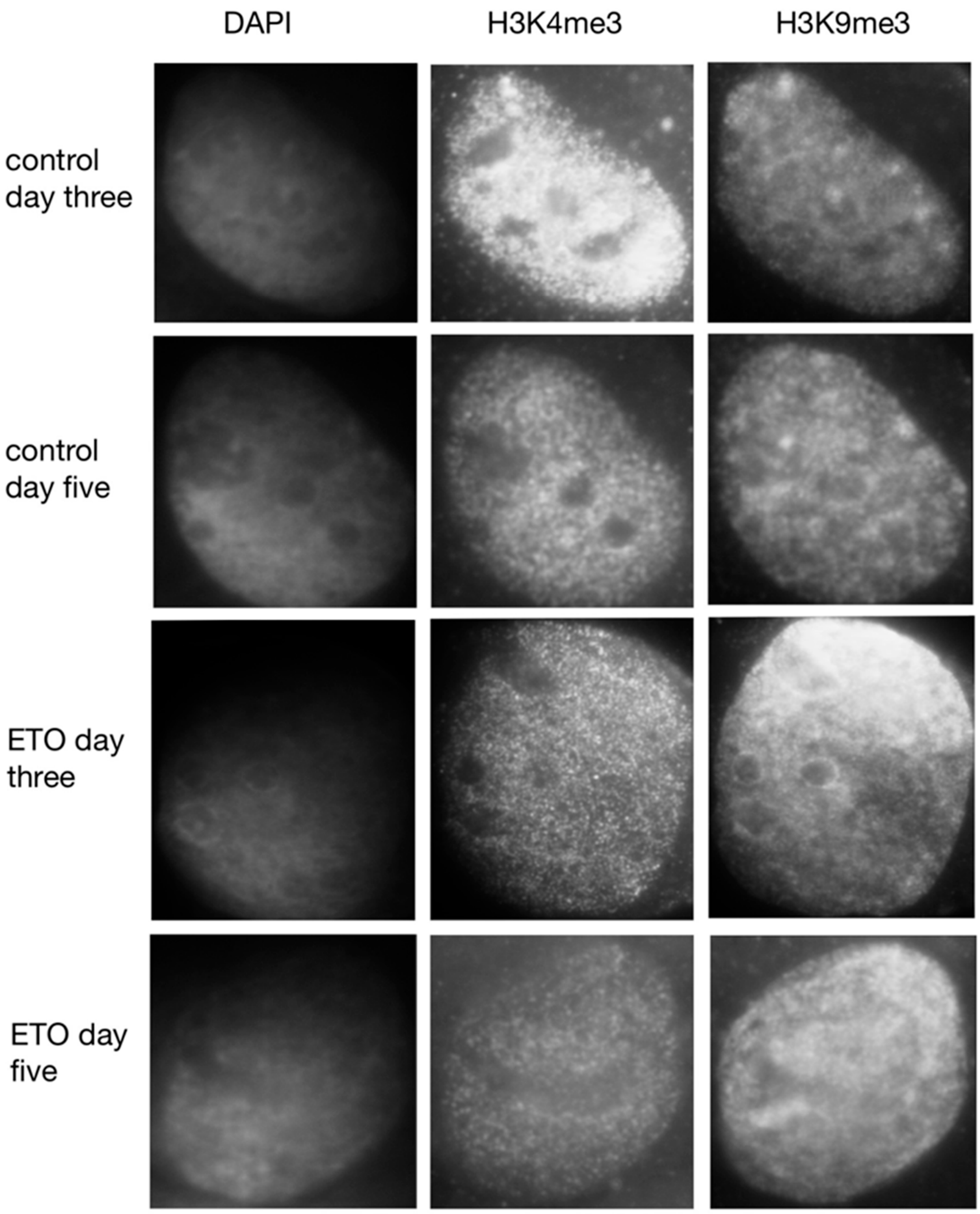

Figure S1: Examples of ETO-treated and non-treated cell nuclei of PA-1 cells on day 3 and 5 after labelling of euchromatin with anti-H3K4me3 antibodies and heterochromatin with anti-H3K9me3 antibodies. While in the non-treated nuclei the chromatin typically shows a granular, network-like distribution, this is more dissolved in the treated cells indicating a loss of the strong separation of eu- and heterochromatin.
